# EDEM3 Domains Cooperate to Perform Its Overall Cell Functioning

**DOI:** 10.3390/ijms22042172

**Published:** 2021-02-22

**Authors:** Georgiana Manica, Simona Ghenea, Cristian V. A. Munteanu, Eliza C. Martin, Cristian Butnaru, Marius Surleac, Gabriela N. Chiritoiu, Petruta R. Alexandru, Andrei-Jose Petrescu, Stefana M. Petrescu

**Affiliations:** 1 Department of Molecular Cell Biology, Institute of Biochemistry, Splaiul Independentei 296, 060031 Bucharest 17, Romania; georgianamanica@gmail.com (G.M.); gheneas@biochim.ro (S.G.); gabic@biochim.ro (G.N.C.); pra@biochim.ro (P.R.A.); 2 Department of Bioinformatics and Structural Biochemistry, Splaiul Independentei 296, 060031 Bucharest 17, Romania; cristian.v.a.munteanu@gmail.com (C.V.A.M.); elizamartinc@yahoo.com (E.C.M.); cristian.butnaru@biochim.ro (C.B.); marius.surleac@biochim.ro (M.S.); ap@biochim.ro (A.-J.P.); 3 Research Institute of the University of Bucharest, 030018 Bucharest 17, Romania

**Keywords:** EDEM3, ERAD, NHK, tyrosinase, mass spectrometry, ER mannosidases, Man1B1, intrinsically disordered domain, protease-associated domain

## Abstract

EDEM3 recognizes and directs misfolded proteins to the ER-associated protein degradation (ERAD) process. EDEM3 was predicted to act as lectin or as a mannosidase because of its homology with the GH47 catalytic domain of the Man1B1, but the contribution of the other regions remained unresolved. Here, we dissect the molecular determinants governing EDEM3 function and its cellular interactions. LC/MS analysis indicates very few stable ER interactors, suggesting EDEM3 availability for transient substrate interactions. Sequence analysis reveals that EDEM3 consists of four consecutive modules defined as GH47, intermediate (IMD), protease-associated (PA), and intrinsically disordered (IDD) domain. Using an EDEM3 knock-out cell line, we expressed EDEM3 and domain deletion mutants to address EDEM3 function. We find that the mannosidase domain provides substrate binding even in the absence of mannose trimming and requires the IMD domain for folding. The PA and IDD domains deletions do not impair the trimming, but specifically modulate the turnover of two misfolded proteins, NHK and the soluble tyrosinase mutant. Hence, we demonstrate that EDEM3 provides a unique ERAD timing to misfolded glycoproteins, not only by its mannose trimming activity, but also by the positive and negative feedback modulated by the protease-associated and intrinsically disordered domain, respectively.

## 1. Introduction

Unfolded and misfolded secretory proteins represent a major burden for endoplasmic reticulum (ER) homeostasis, often leading to cellular stress and conformational diseases [1]. For a protein to attain its native conformation in the ER lumen, it must advance through different maturation steps, like N-glycosylation [2], *cis-trans* prolyl isomerization [3] and disulfide bond formation [4]. As a result, different protein quality control mechanisms are working together to ensure both proper folding and efficient polypeptide disposal. The ER-associated degradation (ERAD) pathway achieves the recognition, retrotranslocation, ubiquitination and proteasomal destruction of proteins that are unable to attain a native state [5,6]. As regards glycoprotein ERAD, it was proposed that the signal for ERAD clearance is generated by the action of α1,2-mannosidases over their attached N-glycans.

In mammals, it was initially postulated that the trimming of only one specific mannose residue by the alpha-1,2 mannosidase Man1B1/ERManI is sufficient to label a substrate for ERAD. The resulting Man_8_GlcNAc_2_ isomer B would have been recognized by ER lectins like protein OS-9 and XTP3B [7]. Successively, they would be transferred back to the cytosol through the retrotranslocon, handed to the ubiquitination machinery and destroyed by the 26S proteasome. Later, it was shown that the extensive trimming leading to Man_5_GlcNAc_2_ or Man_6_GlcNAc_2_ is more likely to be required for proper substrate clearance [8]. Nevertheless, there is still a debate on the molecular mechanism behind this enzymatic interplay, as other members of the glycoside hydrolase 47 family were shown to be active mannosidases [9,10], both in vivo and in vitro. ER degradation-enhancing alpha-mannosidase-like proteins (EDEM1, EDEM2, and EDEM3) are such an example, all working in conjunction with a specific oxidoreductase to fulfill their role [11,12,13]. EDEM2 it is now thought to be the initiator of glycoprotein ERAD [14], by catalyzing the first mannose trimming step, followed by the action of EDEM3 and that of EDEM1 to a lesser extent. Moreover, EDEM2 and EDEM1′s mannosidase activity seems to be dependent on the unfolded state of the glycoprotein substrate [15]. 

However, beside the conserved mannosidase homology domain (GH47), there is little resemblance between the three EDEMs [16], making it difficult to ascertain a general rule for substrate recognition and processing or partner protein interactions. For instance, EDEM3 is a soluble ER protein [10] with an extended structure comprising a protease-associated domain (PF02225) and an ER retention motif (KDEL) at C-terminal. EDEM1 possesses an N-terminal intrinsically disordered domain (IDD) [17] that guides its association with ERAD cargo but not with calnexin or SeL1L. A second C-terminal IDD with potential of facilitating protein-protein interactions [11] has been recently proposed, emphasizing the importance of structural determinants over protein function. 

In this work, we employed a LC/MS approach to detect EDEM3 interactors, along with bioinformatics tools to predict and propose conformational domains for EDEM3. We found that in addition to the GH47 and the PA domains it also possesses an IDD domain that is not vital for protein function. Nevertheless, the PA and IDD domains can guide ERAD substrates processing, acting as positive or negative feedback on protein activity. Moreover, the mannosidase like domain by itself is capable of interacting with misfolded proteins, even though it has no measurable mannose trimming capacity. Finally, EDEM3 collaboration with another known mannosidase Man1B1 resulted in accelerated mannose processing but not in accelerated substrate disposal, bringing new insights into the ERAD landscape. 

## 2. Results

### 2.1. EDEM3 Displays Reduced Stable Interactions with the ER Protein Networks

**Figure 1:**
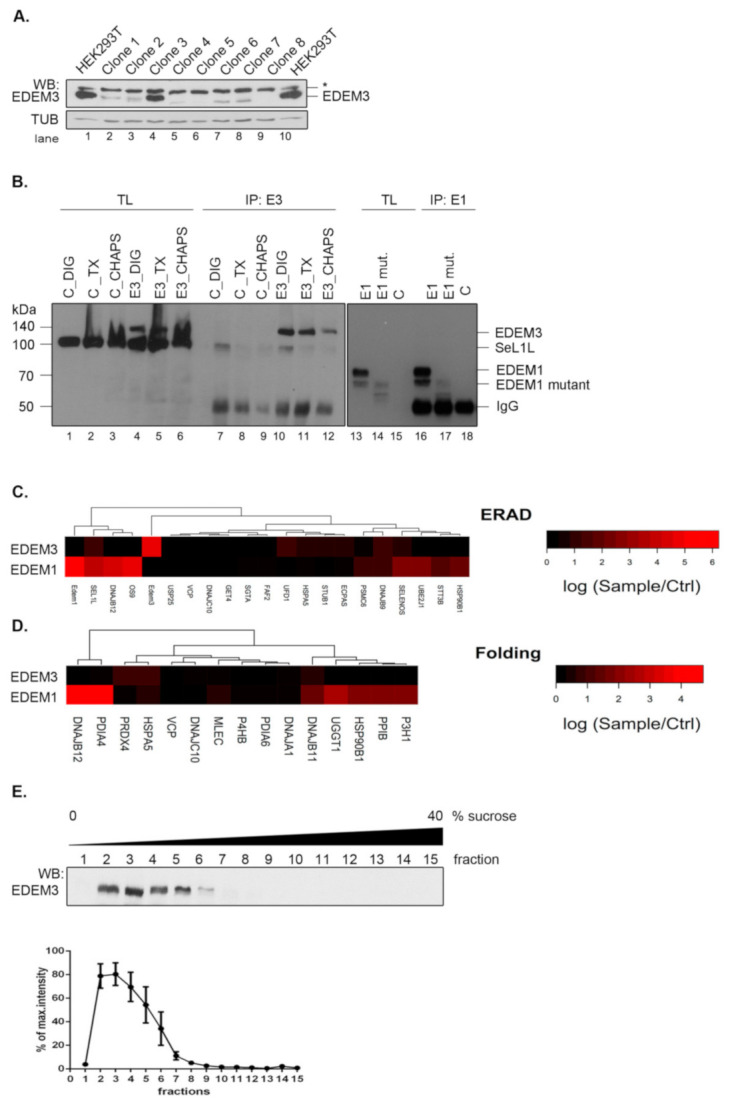
EDEM3 displays a low interaction profile. (**A**) EDEM3-KO cell line clone selection. Western blot analysis for EDEM3 expression in different clones after CRISPR/Cas9 procedure. Tubulin (TUB) was employed as loading control. * denotes unspecific cross-reactivity. (**B**) Pull-down efficiency for EDEM3 and EDEM1 checked by immunoprecipitation and Western blot. The specificity of the EDEM3 antibody was checked in three different lysis buffers, DIG-digitonin, TX-Triton X-100 and CHAPS. EDEM1 and EDEM1-mutant pull-down are also shown for comparison. (**C**) Heat map denoting the spectral counts log ratios of sample/ctrl for the proteins annotated with the ‘ERAD’ key-term in the Gene Ontology Biological Process (GO BP) UniProtKB database. (**D**) Similar with (**C**), but for proteins annotated with the ‘Folding’ key-term and which are ER localized according to GO cellular component (GO CC). For both, only proteins with positive ratios are shown. (**E**) EDEM3 protein distribution on a continuous 0–40% sucrose gradient. Band densitometry of six independent experiments was represented as the mean% from maximum intensity ± SEM.

We have previously reported a potential network of EDEM3 interactors that was highly modulated by the ER stress and the inhibition of the N-glycan processing within the ER [18]. To refine the mass spectrometry analysis, here we took advantage of an EDEM3 knock-out (EDEM3-KO) cell line that allowed a clear-cut distinction between real and false EDEM3 interactors. The EDEM3-KO cell line was generated using HEK293T cells and CRISPR/Cas9 technology. Single cell starting colonies were grown and EDEM3 expression level was tested by Western blotting (Figure 1A). We used a single colony (clone nr.8 in Figure 1A) for all further experiments. To study EDEM3 interactors, the wild-type (WT) protein was subsequently expressed in the EDEM3-KO cells and immunoprecipitated with anti-EDEM3 antibodies. We were able to specifically pull-down EDEM3 using digitonin lysis buffer (Figure 1B, compare lanes 10 and 7). Similar specificity was found for EDEM1 pull -down (Figure 1B, compare lanes 16 and 18). The co-precipitated proteins were separated by SDS-PAGE, trypsinized and analyzed by LC-MS as described in the Materials and Methods section. The results showing the spectral counts log ratios of EDEM3 interactors sample/control are displayed in Figure 1C,D, together with a comparison of EDEM1 interactors.

Analyzing the network of interactors with ER localization annotated as components of the ERAD or folding process, we found that very few proteins co-immunoprecipitate from cell lysates with EDEM3, i.e., SEL1L, HSPA5/BiP, DNAJB9, and UFD1 (ERAD network) (Figure 1C) and HSPA5/BiP, DNAJB11, and PRDX4 (Folding pathway) (Figure 1D). We compared these clusters with those found for EDEM1 pulled–down in the same lysis detergent, displaying similar pull-down specificity towards polyclonal antibodies (Figure 1B) and comparable spectral counts with EDEM3 (Appendix A Table A1). As shown in Figure 1C,D and in a recent report [19], in contrast to EDEM3, the interactors of EDEM1 are abundantly occurring in both ERAD and folding linked networks of proteins. A complementary experiment using protein fractionation by ultracentrifugation (Figure 1E), placed EDEM3 in the lower range of the sucrose gradient fractions. This corresponded to an approximate mass distribution between 120 and 250 kDa, sustaining the diminished interaction profile of the molecule when compared to EDEM1 found in complexes spreading all over the gradient [19]. Importantly, this sedimentation equilibrium predicts the dimerization of the native EDEM3 molecule in the cell. 

The data show that EDEM3 has a relatively low number of stable interactions, which may suggest that in contrast to EDEM1, this GH47 member has a more specialized role in ERAD.

### 2.2. Molecular Organization of EDEM3 Sequence

Within the EDEM family EDEM3 displays the most complex sequence structure. Subjecting it to domain recognition and sequence analysis involving amino acid distribution, accessibility, secondary structure and intrinsic disorder propensities (as described under Materials and Methods) indicate that EDEM3 consists of four distinct structural regions: (a) a glycosidase GH47 domain comprising 501 aa of the overall 931 aa sequence; (b) a ~130 aa region named here for convenience the IMD domain; (c) a protease-associated (PA) domain of ~180 aa; and (d) a final ~130 aa C-terminal region. 

**Figure 2:**
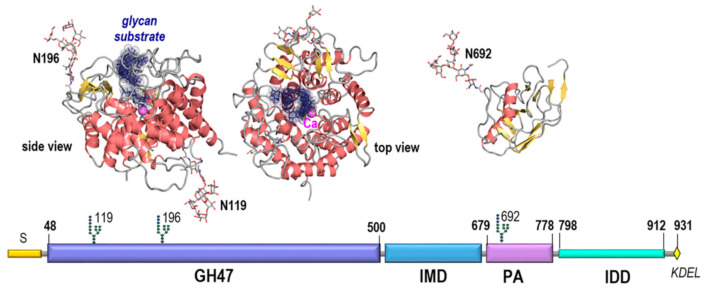
EDEM3 domain organization and display of structural models of GH47 and PA domains. The GH47 model is shown in both side and top views to account for the 3D location of N196- and N119- linked N-glycans.

**Figure A1:**
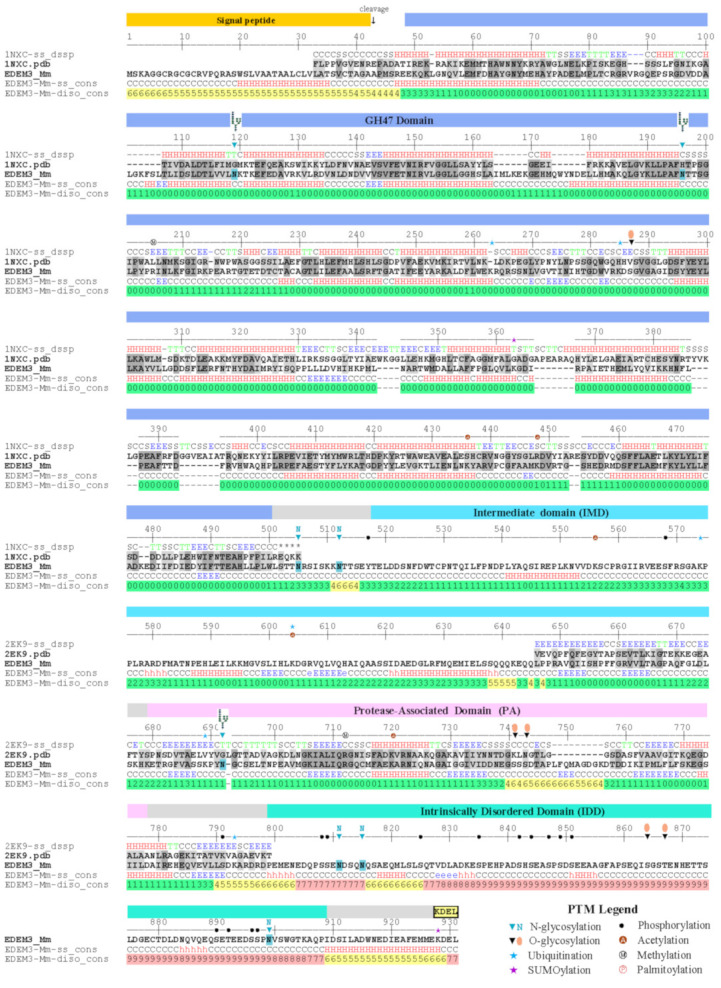
Detailed domain organization, template alignment and structural profiling of EDEM3 sequence. EDEM3 sequence, secondary structure prediction and disorder prediction consensuses are shown on the last three rows, while the templates used in homology modelling are shown above (PDB: 1NXC, 2EK9). Intrinsic disorder propensity is shown on a scale of 0 to 9 (low 0–3 in green, medium 4–6 in yellow, and high 7–9 in red). The legend of the posttranslational modification (PTM) predicted sites (with over 50% probability) is also incorporated in the figure.

Within the overlapping frame, the GH47 domain shares 32% identity with the ER alpha-1,2-mannosidase/Man1B1. Thus, Man1B1 was used to raise a 3D model of the GH47 EDEM3 region as described under Materials and Methods. The global distance test total score and root mean square deviation of the model from an optimal Cα pathway were GDT_TS = 72.3 and RMSD = 2.16Å indicating a very high level of accuracy. As seen from Figure 2 and Figure A1 while the amino acids decorating the large glycan binding site, those involved in catalysis and calcium binding are well conserved between Man1B1 and EDEM3 the pattern of loops interconnecting the secondary structure elements significantly differ by three large insertions and 6 deletions on both sides of the α/α toroid comprising a total 58 amino acid positions. The GH47 model contains two pairs of cysteines with the potential of forming disulfide bridges: C226-C229 and C83-C442. While the first of these is local the second involves distal positions in sequence and was shown to be relevant for activity [13]. In addition the domain contains 2 N-glycosylation sites on opposite sides of the α/α toroid that were both shown to be occupied in mouse and human [20,21].

The sequence profile of the 130 aa stretch separating the GH47 and PA domains indicates that this region is not disordered, nor a proper linker, but rather a regular ordered domain with at least four predicted helices and three beta strands and, therefore, is named here the intermediate (IMD) domain. Fold recognition indicate that the closest template with 15% identity with IMD is the 4rf6B02 region of Arginine kinase 1, pdb code 4rf6 displaying a two-layer sandwich architecture, CATH 3.30. However, as the second structural homologue with 14% identity belongs to a different architecture, CATH 3.90, the structural information at this stage is insufficient to raise a reliable 3D model of this alpha-beta class folded region of EDEM3. 

The IMD is followed by the protease-associated (PA) domain. This is a structural unit with a three-layer sandwich architecture, CATH 3.50.30.30, found in many proteins that was reported to be involved in protein-protein interactions (ppi) [22,23,24]. Fold recognition indicate as closest templates fragments from *Escherichia coli* aminopeptidase and *Spodoptera frugiperda* viral protein, pdb codes 2ek9 and 3kas, with 18% and 14% identities respectively. Using remote homology techniques and a combined piecewise modelling strategy based on the two templates a model of EDEM3 PA domain was raised with GDT_TS=61.34 and RMSD=3.03 Å indicating a high level of accuracy. Both in mouse and human, the PA domain displays the third occupied N-glycosylation site of EDEM3, N692, located in an unusual sequon 692-NGC-694 [20,21]. 

Finally, the C-ter region of EDEM3 sequence, 800–930, displays a very high propensity for intrinsic disorder and therefore was named here the intrinsically disordered domain, IDD. The IDD region displays a highly unbalanced charge distribution with an excess of 35 negatively charged acidic amino acids and a very low density of large hydrophobic amino acids. As can be seen from Figure 2 and Figure A1 there are several short sequence’s stretches predicted to adopt helical structure. Of these the highest propensity is shown by the very C-ter end: IDSILADWNEDIEAFEMMEKDEL containing the ER retention signal. Interestingly the IDD domain shows also the highest density of predicted posttranslational modification sites which also is an indication of the disordered nature of this region.

In summary, modeling and sequence analysis suggests that EDEM3 combines enzymatic and ppi functions performed by distinct modules. The GH47 domain performs glycan trimming while the PA and IDD domains are inferred to be involved in protein-protein interactions. The two functional regions are well separated by the α/β folded IMD domain. In contrast to the PA domain that has well defined structure with large hydrophobic patches on its surface, the IDD domain is flexible and disordered. Intrinsic disordered regions adopt ensembles of configurations as in unfolded protein states [25,26,27]. This allows such regions to morph accordingly to multiple interactors and act as hubs [28,29]. Hence EDEM3 benefits of two distinct mechanisms for binding to its interactors provided by PA and IDD. 

### 2.3. Expression of EDEM3 Mutants in EDEM3 Knock Out Cells

**Figure 3:**
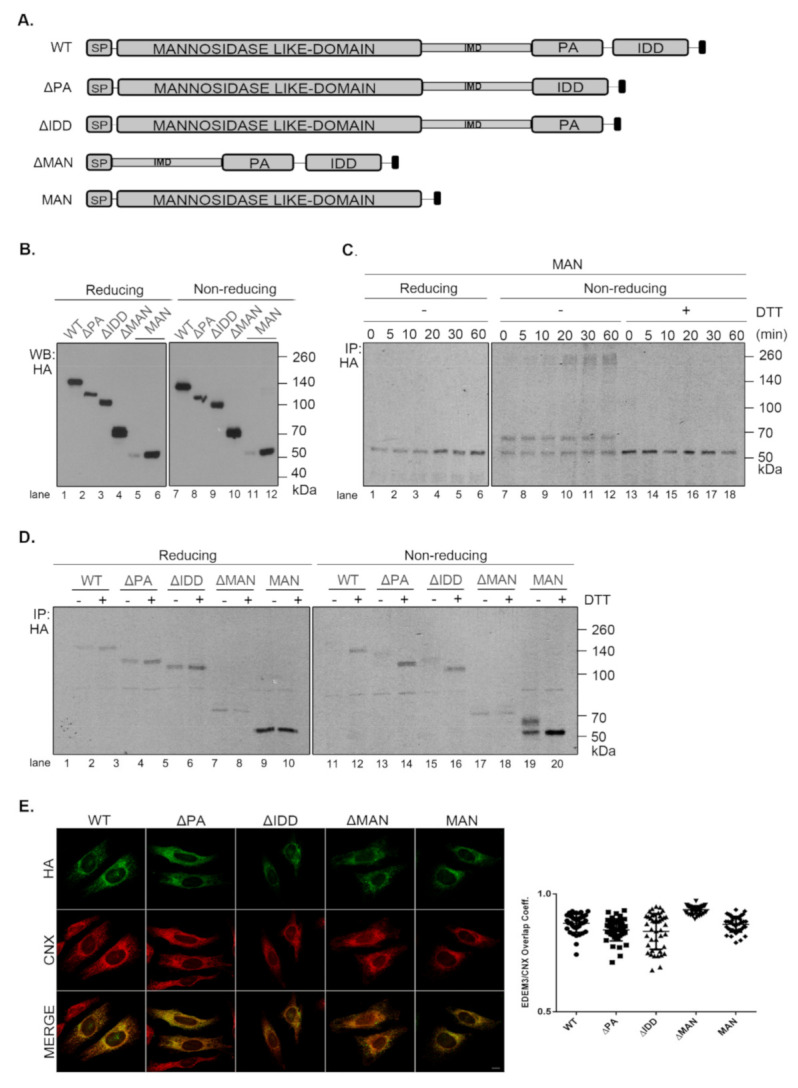
Characterization of EDEM3 constructs. (**A**) Schematic representation of EDEM3 constructs. The SP notation denotes the signal peptide, while the black squares represent the KDEL. (**B**) EDEM3 constructs expression levels in EDEM3-KO cell line. Equal amounts of protein lysates were separated in reducing and non-reducing conditions and the expression level was tested by Western blotting. Given the low signal obtained for MAN (lane 5, 11) the DNA quantity used for transfection was doubled (lane 6, 12). (**C**) Redox sensitivity of the MAN construct determined by pulse chase analysis. DTT was added to the cell media to modulate the folding of the protein during the indicated time points. The immunocomplexes were separated in reducing and non-reducing conditions by SDS-PAGE and the results were visualized by autoradiography. (**D**) Same as in (**C**), except the cells were transfected to overexpress WT and all other EDEM3 mutants and harvested immediately after the pulse. (**E**) Colocalization of EDEM3 mutants with calnexin. HeLa cells were transfected with the EDEM3 mutants and processed for immunofluorescence with mouse monoclonal anti-HA and rabbit anti-calnexin (CNX) antibodies. The overlap coefficient was determined for 40 individual cells and represented as (mean ± SD).

**Figure A2:**
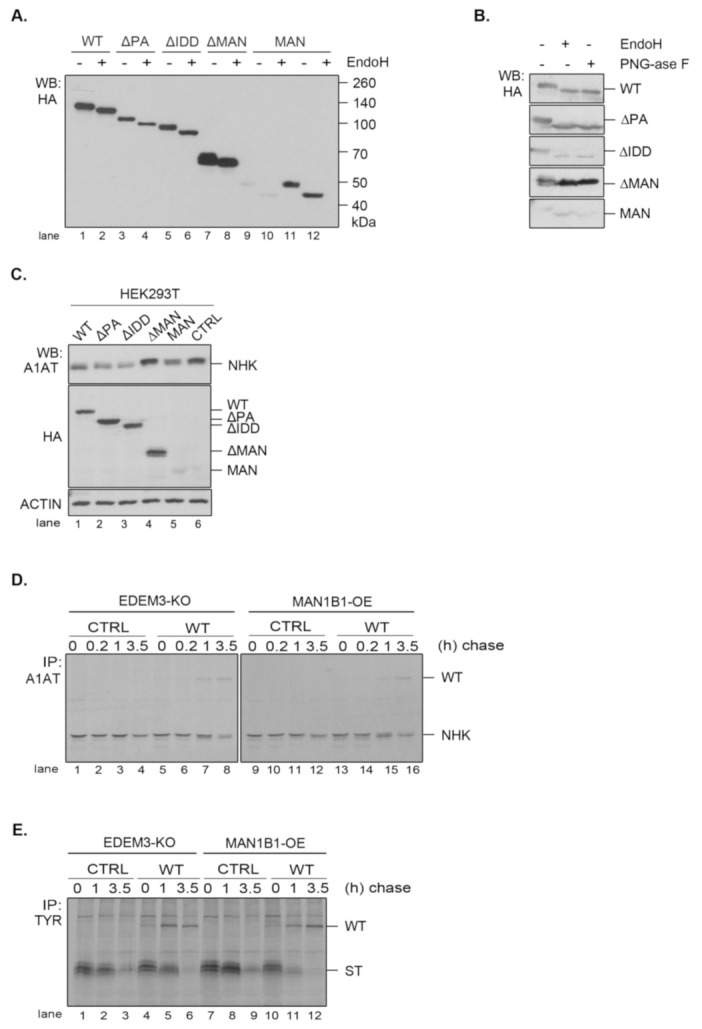
(**A**) EDEM3 constructs are processed by EndoH. EDEM3-KO cells were transfected with plasmids encoding for EDEM3 constructs. Lysates were treated with EndoH and subjected to Western blotting. (**B**) Same as in (**A**), except lysates were also incubated with PNG-aseF. (**C**) Western bloting analysis of NHK processing by EDEM3 constructs in HEK293T cell line. After co-transfection with NHK and EDEM3 constructs, cells were lysed and processed for WB. Rabbit anti-A1AT and rat anti-HA antibodies were used for detection. Actin was used as loading control. (**D**) NHK intracellular processing in EDEM3-KO and MAN1B1-OE cells. Following co-transfection with the appropriate plasmids, cells were starved, pulse labeled and chased for the indicated time points. Immunoprecipitation was performed using rabbit anti-A1AT antibodies. Resulting immunocomplexes were separated by SDS-PAGE and results were visualized by autoradiography. (**E**) Same as in (**D**) with the exception that ST was used.

In order to better understand the role of EDEM3 domains, we generated a series of truncated mutants of EDEM3 (Figure 3A). We sequentially deleted the main conformational domains predicted by the bioinformatics analysis and obtained four distinct mutated proteins, as follows: one lacking the protease-associated domain (ΔPA), one lacking the intrinsically disordered domain (ΔIDD), one containing the C-terminal portion of the protein, thereby lacking the mannosidase-like domain (ΔMAN), and one containing only the GH47, named here the mannosidase-like domain (MAN). The signal peptide and KDEL sequence were maintained in all mutants. These constructs were transiently expressed in the EDEM3-KO cell line and found to migrate at steady state at their predicted molecular masses, considering the predicted post-translational modifications (Figure 3B). The more abundant ΔMAN mutant displays two individual bands, corresponding to a glycosylated and to a non-glycosylated species (Figure A2A, lanes 7, 8). The MAN mutant was better detected in Western blot at higher DNA concentrations (Figure 3B, lanes 5, 11 vs. lanes 6, 12) and further recognized by immunoprecipitation from pulse-labelled cells (Figure 3C). In non-reducing conditions (Figure 3B, right panel), all EDEMs migrated as monomers with WT, ΔPA, and ΔIDD showing slightly higher mobilities than their reducing counterparts.

To begin to understand the role of the multiple domains in EDEM3 folding and maturation, we analyzed the redox sensitivity of the native disulfide bridges of the MAN mutant. Thus, pulse-labeled cells were chased in the presence of low concentrations of DTT in the cell media (5mM DTT) to reduce only non-buried disulfide bridges. The cell lysates were immunoprecipitated with anti-HA antibody and analyzed in reducing and non-reducing SDS-PAGE (Figure 3C). In non-reducing conditions, at all chase points, the MAN mutant showed two bands, a slower migrating DTT-sensitive adduct and a faster migrating fully reduced monomeric band. At the late chase points, more species with higher molecular masses were detected. When DTT was present in the cell (Figure 3C, lanes 13–18) and in reducing conditions (Figure 3C, lanes 1–6), the high molecular mass species were no longer recovered at any chase point. This suggests that they represent disulfide cross-linked complexes of the newly synthesized MAN domain. 

We further compared all the EDEM3 mutants in a pulse-chase experiment analyzing the first chase point (Figure 3D). We found that WT, ΔPA and ΔIDD mutants displayed a DTT-sensitive adduct as a very diffuse band in non-reducing conditions. In the presence of DTT in the cell, or in reducing gels, these mutants migrate with higher mobilities, as sharp bands of fully reduced monomers (Figure 3D, non-reducing lanes 12, 14, 16, and reducing, lanes 1–6). In contrast, but confirming the results in Figure 3C, the MAN mutant migrates as two bands that are reduced to one in the presence of intracellular DTT (Figure 3D, lane 20) and in reducing gels (Figure 3D, lanes 9, 10). Only the ΔMAN mutant was completely insensitive to the DTT treatment (Figure 3D, lanes 17, 18), indicating that in the absence of the mannosidase-like domain there are no sensitive disulfide bridges. 

The presence of a KDEL sequence dictates the endoplasmic reticulum (ER) retention of a protein. Indeed, all EDEM3 proteins were sensitive to EndoH (Figure A2A) and PNGase-F (Figure A2B) with the de-glycosylated polypeptides migrating at similar molecular masses. This is indicative of an oligomannosidic structure for the covalently linked N-glycans corresponding to ER resident proteins [30]. Moreover, in immunofluorescence microscopy, all mutants co-localize with the ER-resident protein, calnexin (Figure 3E), with high overlap co-localization coefficients (Figure 3E, quantification). We therefore have generated EDEM3 mutants lacking the main domain of the wild type protein whilst maintaining their stable expression and ER localization. Taken together, these results also show that EDEM3 forms adducts linked through disulfide bridges through the GH47 domain that could play an active role in its function.

### 2.4. EDEM3 Mutants Show Differential Effects on the Degradation of the Misfolded Soluble Tyrosinase Mutant

**Figure 4:**
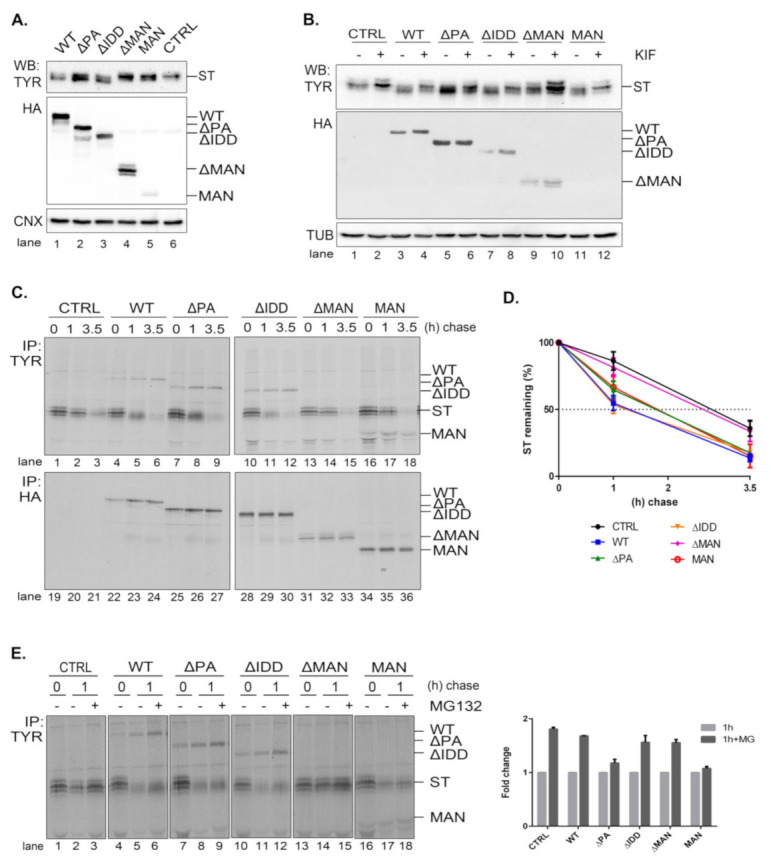
Degradation of soluble tyrosinase in the presence of EDEM3 mutants. (**A**) Soluble tyrosinase (ST) is processed differently by EDEM3 constructs. EDEM3-KO cells were co-transfected with plasmids encoding for soluble tyrosinase (ST) and EDEM3 mutants and processed for Western blotting with polyclonal anti-tyrosinase (TYR) antibodies and monoclonal anti-HA. Calnexin (CNX) was used as loading control. (**B**) Kifunensine impairs mannosidase properties of EDEM3 constructs. Same as in (**A**) except cells were treated with 25 µM kifunensine for 16h prior to harvesting. Tubulin (TUB) was used as loading control. (**C**) ST degradation rate is modulated by EDEM3 mutant proteins. EDEM3-KO cells were co-transfected with plasmids encoding for ST and EDEM3 constructs and subjected to pulse-chase analysis. Half of cell lysate was incubated ON with HM anti-tyrosinase (TYR) antibodies (upper panel) while the other half was incubated with mouse anti-HA antibody (lower panel). The resulting immunocomplexes were separated by SDS-PAGE. Bands were visualized by autoradiography. (**D**) Graphic representation of ST degradation rate from (**C**) as (mean ± SD) of three independent experiments. (**E**) Proteasomal degradation of ST is fine-tuned by EDEM3 constructs. Same as in ((**C**), upper panel), except cells were treated with 10 µM MG132 for 1 h prior to immunolabeling. Band densitometry quantification of treated condition versus non-treated at 1 h chase is presented as the mean ± SEM of two independent experiments.

A soluble form of tyrosinase (ST) is an established ERAD substrate [17,31,32] that we further used to characterize the role of the EDEM3 mutants in its traffic and disposal. Knowing that EDEM3 is an active mannosidase, both in vitro and in vivo, we interrogated the role of each functional domain in ST processing. We co-transfected the corresponding plasmids in the EDEM3-KO cell line and proceeded for Western blotting (Figure 4A). As expected, WT and EDEM3 mutants possessing the mannosidase-like domain, namely ΔPA and ΔIDD, were capable of enzymatic trimming, a faster migrating species of ST appearing at steady state. In contrast, ΔMAN lost its activity, suggesting that the mannosidase-like domain is indeed the one responsible for substrate handling. On the other hand, the results for MAN were quite ambiguous, there being a small residual activity when compared to the empty vector transfection. In order to clarify which of the EDEM3 constructs still maintains mannosidase activity, we treated cells ON with 25 µM kifunensine (Figure 4B) and performed the same protocol. It became obvious that the PA and IDD domains did not influence the enzymatic properties of EDEM3, while the ΔMAN construct lost all enzymatic capacity. A slight mass shift was observed for ST in the presence of MAN when treated with kifunensine, suggesting that the mannosidase-like domain cannot conserve by itself the mannosidase activity of WT. Further, we studied the effect of EDEM3 mutants upon ST degradation. The corresponding plasmids were co-transfected in the EDEM3-KO cell line. Cells were starved, pulsed and chased for the indicated time periods (Figure 4C). Half of the cell lysate was incubated with anti-tyrosinase antibodies while the other half with anti HA-antibodies. As expected, WT decreased ST half-life by approximately 1.5 h (Figure 4D), whilst ΔMAN did not influence the ST clearing. Deletion of IDD did not prove to be crucial for ST accelerated degradation, contrary to what was observed for EDEM1 [17]. However, the loss of the PA or MAN domain resulted in an almost 30 min lag in ST half-life when compared to WT. Importantly, both mutants were able to accelerate the degradation of ST, albeit to a reduced level as compared to WT. A clear mass shift of ST occurred during chase upon EDEM3 overexpression, but no shift could be detected in the presence of ΔMAN. Thus, the MAN mutant was active in the ERAD of the tyrosinase mutant, despite the considerable loss of its mannosidase function. 

It is known that EDEM3 induced degradation is dependent on the proteasome function. To verify whether EDEM3 constructs direct the substrate degradation towards proteasomes, we performed a pulse-chase and immunoprecipitation experiment in the presence of the proteasome inhibitor MG132 (Figure 4E). Indeed, we found an accumulation of ST at 1 h chase for all EDEM3 mutants. Interestingly, the two mutants lacking the PA domain (ΔPA and MAN) were less sensitive to the proteasomal inhibition (Figure 4E, quantification), indicating a role for the PA domain in facilitating the proteasomal degradation of ERAD substrates. 

Taken together, the data show that EDEM3 and most of its mutants accelerate the degradation of the soluble tyrosinase mutant, excepting for the ΔMANmutant, defective in the GH47 domain. Moreover, the mutants lacking the PA domain have a reduced effect upon ST degradation, with a specific influence upon its proteasome degradation pathway. 

### 2.5. The PA and IDD Domains of EDEM3 Are Not Required for the Accelerated Mannose Trimming of the Misfolded Null Hong Kong α1-Antitrypsin

**Figure 5:**
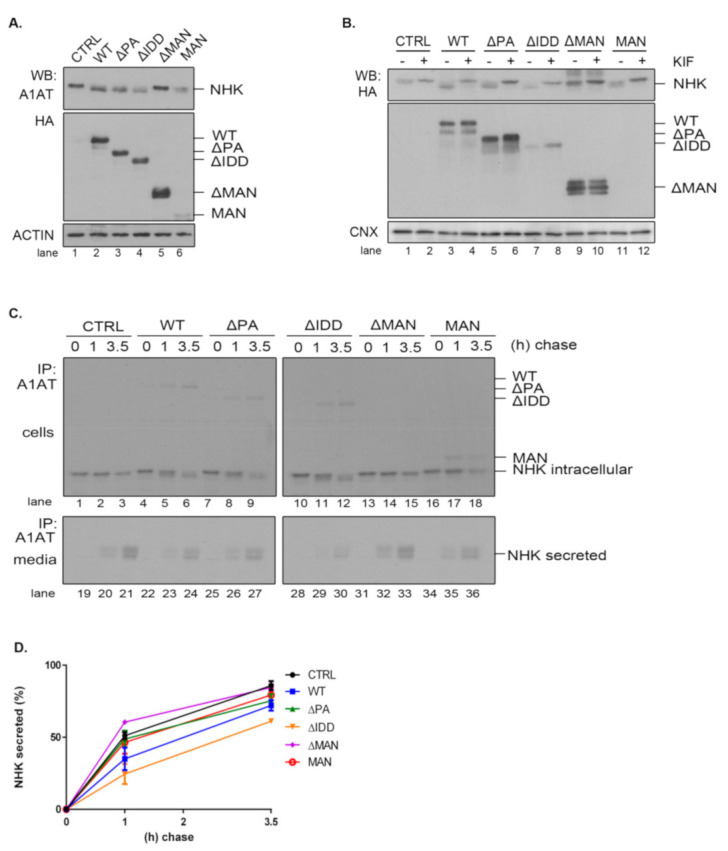
NHK mannose trimming in the presence of EDEM3 mutants. (**A**) NHK processing by the EDEM3 constructs. EDEM3-KO cells were co-transfected with NHK and EDEM3 mutants and processed for Western blotting. The resulting membranes were incubated with rabbit anti-α-1 antitrypsin (A1AT), rat anti-HA and mouse anti-actin antibodies. (**B**) Kifunensine treatment hinders EDEM3 constructs mannosidase properties. EDEM3-KO cells were co-transfected with EDEM3 constructs and NHK. Kifunensine (25 µM) was added to the cell media and equal aliquots of cell lysates were separated by SDS-PAGE and processed for WB with rat anti-HA antibody. Calnexin (CNX) was used as loading control. (**C**) Pulse-chase analysis of NHK intra and extracellular fate. EDEM3-KO cells were co-transfected with plasmids encoding for NHK and EDEM3 constructs. After immunolabeling, lysates (upper panel) and cell media (lower panel) were immunoprecipitated with rabbit anti-A1AT antibodies ON. The isolated immunocomplexes were separated by SDS-PAGE and visualized by autoradiography. (**D**) Graphic representation of NHK secretion rate as percent of total amount of three independent experiments (mean ± SEM).

Given the diversity of ERAD substrates, we have chosen the null Hong Kong α1-antitrypsin mutant (NHK) as another suitable molecule to inquire the mannosidase trimming of EDEM3 constructs. We used the same approach as for ST. For this reason, EDEM3-KO cells were co-transfected for 48 h with plasmids encoding for both EDEM3 and NHK and analyzed by SDS-PAGE followed by Western blotting (Figure 5A). Again, we were able to distinguish between a definite mannosidase activity for WT, ΔPA, and ΔIDD, no activity for ΔMAN, and poor activity at steady state for MAN. Similarly, when the constructs were expressed in HEK293T cells together with NHK, the trimming activity of the MAN mutant was abolished (Figure A2C). These findings were confirmed after treating cells overexpressing NHK and EDEM3 mutants with kifunensine (Figure 5B). By blocking the mannosidase activity of the EDEM3 constructs, we reversed the mass shift for NHK in the presence of WT, ΔPA, and ΔIDD. For the ΔMAN construct on the other hand, the mobility of NHK was not influenced by the addition of the inhibitor, whilst the MAN mutant showed an incremental mobility shift in the presence of kifunensine. Further, we investigated the NHK processing by pulse-chase and immunoprecipitation. Cells were starved, pulsed with [^35^S] cysteine/methionine, and chased for the indicated time points (Figure 5C). Cell lysates and cell media were immunoprecipitated with anti-alpha-1 antitrypsin (A1AT) antibodies. Immunocomplexes were captured on protein A/Sepharose, eluted and subjected to SDS-PAGE. As found for ST, co-expression of NHK and WT resulted in a significant trimming of NHK N-glycans even after one hour of chase (Figure 5C, lane 2 vs. lane 5). Deletion of the PA or IDD domains did not modify this accelerated trimming, supporting the idea that these domains are redundant for the enzymatic activity of EDEM3. As expected, deletion of the mannosidase-like domain resulted in the loss of the enzymatic activity (Figure 5C, lanes 13–15). Interestingly, there was no noticeable mass shift for NHK in the presence of the MAN mutant. The residual trimming activity of the MAN mutant found merely at steady state, suggests that the mannosidase domain favors the exposure of the substrate to additional trimming coming possibly from the other mannosidases. 

Even if NHK is a misfolded mutant, within a recombinant system the degradation capacity of the ER could be exceeded and NHK molecules would be secreted in the media [33]. Investigating the NHK secretion, we found that the levels reached by the ERAD substrate in the cell media are in agreement with EDEM3 constructs activity (Figure 5C, lower panel and Figure 5D). Thus, WT and the ΔIDD mutant diminish NHK secretion rate when compared to the mock transfer, with the ΔIDD mutant being the most effective. Also, ΔPA and MAN mutants are able to retain NHK in the ER, lowering the NHK amount available for secretion. Solely the ΔMAN mutant permits NHK secretion at the same extent as the mock transfer. 

Taken together, the data show that PA and IDD domains are not required, per se, for EDEM3 activity, with the mannosidase-like domain being the one responsible for the mannose trimming. The IDD domain negatively regulates NHK secretion by inhibiting its ER retention. However, in the absence of the rest of the molecule, the mannosidase-like domain cannot preserve its enzymatic properties, although it seems able to retain substrates within the ER.

### 2.6. EDEM3 Is Both a Mannose Trimming Enzyme and a Lectin-Like Protein 

**Figure 6:**
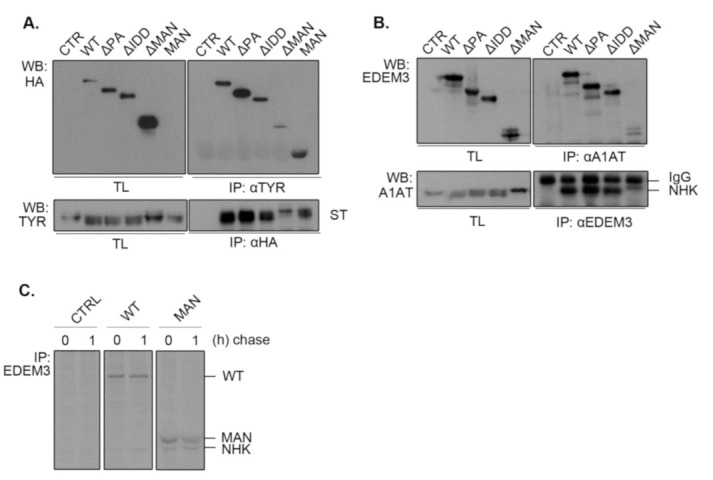
Interaction of EDEM3 mutants with NHK and soluble tyrosinase. (**A**) Analysis of ST association with EDEM3 constructs. Co-transfected lysed cells were divided in two: one half was immunoprecipitated with anti-tyrosinase antibodies while the other was immunoprecipitated with anti-HA antibodies. Immunoblots of isolated immunocomplexes were visualized with the indicated antibodies. TL indicates total lysates. (**B**) Analysis of NHK association with EDEM3 constructs. Cells were co-transfected with NHK and EDEM3 plasmids and processed as in (**A**), with the exception that anti-alpha-1 antitrypsin (A1AT) and anti-EDEM3 antibodies were used. TL indicates total lysates. (**C**) Analysis of MAN construct association with NHK. Due to the fact that commercial antibody used in (**B**) does not recognize MAN-EDEM3, an EDEM3 home-made antisera was used instead for immunoprecipitation after cell pulse labeling.

Considering that the mannosidase-like domain was shown to associate with misfolded glycoproteins covalently [13], we investigated whether the deletion of other functional domains impairs EDEM3 association with our ERAD substrates. For this, we co-expressed ST (Figure 6A) or NHK (Figure 6B) along with EDEM3 mutants in the EDEM3-KO cell line for 48h. Cell lysates were divided in two: one part was incubated with anti-substrate antibodies, while the other was incubated with anti-HA or anti-EDEM3 antibodies. The obtained immunocomplexes were subjected to SDS-PAGE and Western blotting. Finally, the membranes were probed with the mentioned antibodies. For both ST and NHK, we observed a strong association between the misfolded proteins and WT, ΔPA, and ΔIDD, concurring with their enzymatic and degradative properties. By contrast, deletion of the mannosidase-like domain produced low to none association between the two investigated species. Interestingly, the MAN mutant associated with ST as well (Figure 6A), suggesting that this domain is indeed responsible for the substrate targeting. Unfortunately, when studying NHK and MAN association, the used commercial anti-EDEM3 antibodies did not recognize MAN. Therefore, we changed the experimental approach and performed a pulse chase and immunoprecipitation experiment using a home-made anti-EDEM3 antibody (Figure 6C). As found also during the previous pulse-chase experiments (Figure 5C), NHK co-immunoprecipitates with the MAN mutant at both chase points. Nevertheless, in the pulse-chase experiments from Figure 4 and Figure 5, we could observe a different association pattern for WT, ΔPA, and ΔIDD versus MAN mutant. The first three were increasingly co-immunnoprecipitated with either ST or NHK during the chase, reaching the highest level at 3,5h chase point. By contrast, the MAN mutant reached a maximum co-precipitation at 1h chase and then decreases in intensity. These show a preferential association of EDEM3 with trimmed substrates which indicates that EDEM3 can function both as an enzyme to catalyze the substrate mannose trimming and as a lectin with specificity for its own enzymatic product. 

### 2.7. EDEM3 and ERManI/Man1B1 Act Together in the Mannose Trimming Rather than in the ERAD Substrates Degradation

**Figure 7:**
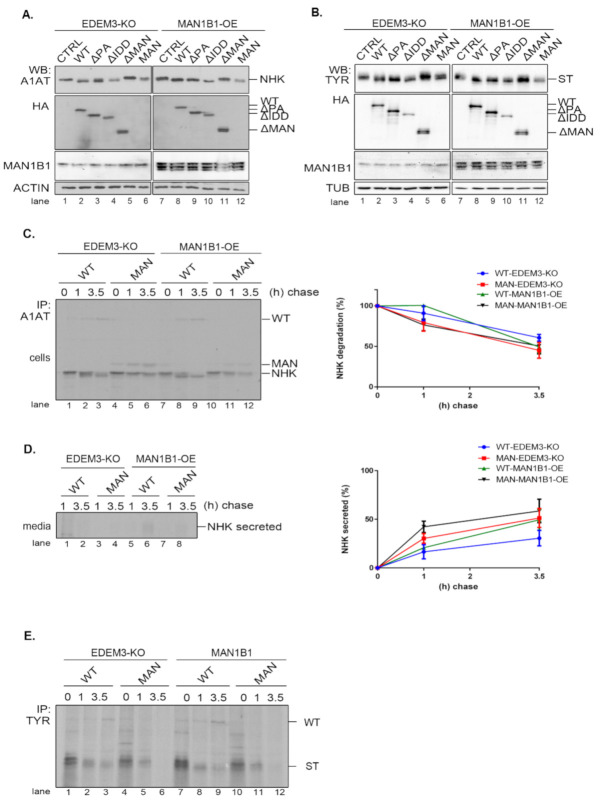
EDEM3 function in the presence of Man1B1. (**A**) Western blot comparison of NHK processing by EDEM3 constructs in EDEM3-KO and MAN1B1-OE cell lines. After co-transfection of indicated plasmids, cell lysates were separated by SDS-PAGE and processed for WB with rabbit anti-A1AT, rat anti-HA, mouse anti-MAN1B1 and mouse anti-actin antibodies. (**B**) Western blot comparison of ST processing by EDEM3 constructs in EDEM3-KO and MAN1B1-OE cell lines. Cells were co-transfected with corresponding plasmids and analyzed by WB with mouse anti-TYR, rat anti-HA, mouse anti-MAN1B1. Rat anti-tubulin (TUB) were used as loading control. (**C**) Immunolabeling analysis of intracellular NHK in EDEM3-KO and MAN1B1-OE cell lines. Cells were co-transfected with NHK and WT or MAN plasmids and they were pulse-labeled accordingly. After immunoprecipitation, the isolated complexes were separated by SDS-PAGE and results were visualized by autoradiography. Graphic representation of NHK degradation rate is presented as (mean ± SEM) of 3 independent experiments. (**D**) Extracellular fate of NHK in EDEM3-KO and MAN1B1-OE cells. Collected cell media from (**C**) was processed for immunoprecipitation with anti-A1AT antibodies and the obtained immunocomplexes were separated by SDS-PAGE. NHK secretion rate is represented as (mean ± SEM) of three independent experiments. (**E**) ST processing by WT and MAN in EDEM3-KO and MAN1B1-OE cell lines. Cells were immunolabeled as described and subjected to immunoprecipitation with rabbit anti-tyrosinase (TYR) antibodies. Immunocomplexes were separated by SDS-PAGE and the results were visualized by autoradiography.

As EDEM3′s high mannose processing seems to be its primary function, we investigated whether supplementary trimming would improve substrate disposal. For this, we generated a HEK293T cell line overexpressing the Man1B1 (MAN1B1-OE). The EDEM3 mutants, alongside NHK or ST, were co-expressed in both EDEM3-KO and MAN1B1-OE cell lines and analyzed by WB. As depicted in Figure 7A,B, there is some cooperation between the two wild type mannosidases as the processed NHK (Figure 7A, lane 2 vs. lane 8) and ST (Figure 7B, lane 2 vs. lane 8) migrate faster as sharper bands in SDS-PAGE. The added trimming effect was maintained for all enzymatically active mutants, whilst for ΔMAN and MAN there was no identifiable additional NHK trimming. Actually, we were able to point that the simultaneous presence of WT-EDEM3 and Man1B1 promoted the appearance of faster migrating form of NHK (Figure 7C, lane 2 vs. lane 8). Unexpectedly, the accelerated trimming was not necessarily equivalent with accelerated NHK degradation (Figure 7C, quantification), but rather with accelerated NHK secretion. This was not statistically significant, but there was a clear secretion trend (Figure 7D). Moreover, the lack of mannosidase activity of the MAN mutant impeded NHK processing even upon Man1B1 overexpression (Figure 7C, lanes 10–12). In fact, when the MAN mutant was replaced with an empty vector (Figure A2D), the NHK processing was similar, indicating that Man1B1 may require input from other ER mannosidases for its activity. Likewise, pulse-chase experiments using the ST substrate instead of NHK, show that the degradation trend of ST is not accelerated by the overexpression of Man1B1 together with EDEM3 or its MAN mutant (Figure 7E). Moreover, Man1B1 is unable to improve the mannose trimming of ST in the presence of the catalytic inactive MAN mutant (Figure 7E, lanes 10–12), as occurred in the case of a mock transfer (Figure A2E). 

Taken together, these results suggest that overexpression of EDEM3 and Man1B1 yields an improved trimming of the ERAD substrates NHK and ST, but this does not result in an accelerated degradation of the misfolded substrates. However, the co-upregulation of the two mannosidases could impair the retrograde transport of NHK from the KDEL receptor back to the ER and facilitate its secretion.

## 3. Discussion

EDEM3 has the most complex structure among the EDEM proteins. Sequence analysis indicates that subsequent to the common GH47 domain, EDEM3 has three successive modules: IMD, PA, and IDD. Of these, the PA and IDD were shown in other systems to be involved in protein-protein interactions (ppi) by two distinct mechanisms. While the PA interacts via surface complementarity as being folded with a well-defined architecture [23] the IDD is intrinsically disordered and prone for multiple transitory ppi interactions [28,29]. In this way the N-glycan processing unit, at the N-terminal end of EDEM3, is neatly separated via the folded IMD domain from the protein-protein interaction modules found toward the C-ter end of the protein. Though unexpectedly, the MS analysis of EDEM3 interactors showed infrequent stable interactions of EDEM3 with other ERAD components or with folding machinery proteins. This is in startling contrast with EDEM1, its closest related member of the EDEM family, which was shown to participate in various ER quality control complexes within the ER [19]. By contrast, ultracentrifugation experiments place EDEM3 at molecular masses varying between 120–250 kDa, indicating its functioning as a homo- or hetero-dimer within the ER. Our data indicate that EDEM3 has a more limited function in the final steps of the ERAD process than EDEM1. Based on the results presented here, we propose that EDEM3 acts upon misfolded glycoproteins as a mannosidase/lectin rather than an ERAD component able to handle the substrate to the SEl1L complex for degradation. Indeed, the study of EDEM3 knock-out cells allowed the detection of the mannose trimming of the two ERAD substrates investigated here merely upon restoring the expression of EDEM3. This is also in accordance with previous reports on the misfolded proteins NHK and TCRα, showing the mannose trimming activity of EDEM3 [10,13]. Recently, EDEM3 was shown to possess mannosidase activity in vitro [13], which reinforce it as a highly active ER mannosidase.

To get insights into the specific roles of the EDEM3 domains for its biological function, we tested domain deletion mutants designed to enter and be retained within the ER. We found that in the absence of the mannosidase-like domain, EDEM3 is catalytically inactive and unable to accelerate the degradation of the ERAD substrates: the misfolded tyrosinase and NHK. In addition, the absence of the mannosidase-like domain also abolished the interaction with the misfolded proteins. This is interesting since, in the case of EDEM1, the inactivation of the active mannosidase center or the deletion of most of the domain, did not impair either the association with the ERAD substrate or the accelerated degradation effect [34,35]. EDEM1 has an intrinsically disordered domain shown to interact with the misfolded substrates but also with the ERAD components [17,19]. However, in the case of EDEM3, the mutant lacking the intrinsically disordered domain, ΔIDD, is not only enzymatically active, but also able to interact with and efficiently target both ST and NHK to degradation. For NHK, this domain appears to confer EDEM3 additional control upon selected ERAD substrates, as in its absence more NHK molecules are retained in the ER and not secreted. It is likely that the IDD prevents some interactions, thus conferring a negative feedback for EDEM3 modulated degradation. 

Similar to the IDD domain, the PA domain is dispensable for the mannose trimming activity of EDEM3 and for the association and targeting to degradation of misfolded ST and NHK substrates. However, in the particular case of the tyrosinase mutant, the PA domain is required to maintain the higher turnover imposed by EDEM3 at the level of the wild type EDEM3. Therefore, we begin to understand that the IDD and PA domains play subtle roles in the modulation of EDEM3 degradation pathway that are substrate specific, as PA interferes with EDEM3 function for misfolded tyrosinase, whilst IDD mostly for the NHK degradation. 

In the absence of the IMD adjacent domain, the mannosidase-like domain is unable to perform its enzymatic function and thus the ERAD of the misfolded proteins is not enhanced. Whereas the mutant is stably expressed in the EDEM3-KO cells, there is a discrepancy between the folding intermediates of this mutant as compared with the others. Thus, in non-reducing conditions, the MAN mutant displays besides the monomer, several higher molecular mass mixed disulfides. In contrast, the wild-type and ΔPA and ΔIDD mutants migrate solely as one DTT-sensitive adduct, indicating that mannosidase-like domain adopts a different conformation that prevents the formation of all native disulfides. These data are supported by a previous report showing that EDEM3 activity depends on its redox status [13]. Whether the transiently forms species are homo- or hetero-dimers with PDI-like proteins from the ER, such as Erp46, as previously found, we could not confirm. However, in ultracentrifugation experiments, EDEM3 sediments as a hetero- or homo-dimer at approximately 120–250 kDa, hence we propose that this DTT-sensitive dimer may play an important role in EDEM3 function and regulate the redox properties of the molecule. Indeed, in the absence of a native conformation, most of the MAN mutant molecules are unable to dimerize, resulting in a catalytically inactive mutant. Further experiments are required to fully understand the molecular nature of this EDEM3 adduct.

We should note however that the mannosidase-like domain retains its capacity of binding to both canonic ERAD substrates. Considering that only mutants containing the mannosidase-like domain interact with the two misfolded proteins, we demonstrate that the association with the substrate occurs through the mannosidase-like domain. Interestingly, in the absence of mannose trimming activity, this mutant interacts for shorter periods of time with the substrate, as shown in pulse chase experiments of soluble tyrosinase and of NHK (Figure 4C, upper lane and Figure 5C, compare WT with MAN mutant co-immunoprecipitation during chase). This may be due to the fact that the trimmed glycoproteins are released form calnexin and available for EDEM3 binding, as shown previously for EDEM1 [36,37]. Alternatively, EDEM3 mediated retention of the substrate may require a preliminary mannose trimming to allow the lectin type association. Therefore, related to the long debate on whether EDEMs are mannosidases or lectins [16], we propose that EDEM3 is both a mannosidase and a lectin acting to prolong the ER retention of the terminally misfolded polypeptides before their presentation to the OS9 and XTP3B Man5-6 lectins.

Finally, we found an unexpected effect of the simultaneous overexpression of EDEM3 and ERMANI/Man1B1. As previously suggested [38,39], the two mannosidases display an advanced mannose trimming of the misfolded tyrosinase and NHK proteins, as compared with the single expression of EDEM3. Indeed, Man1B1 was initially reported to be the first mannosidase within the ER that trims the glycan from M9 to M8 oligomannoses and those were further recognized by EDEM1-3 and targeted for degradation. Following reports demonstrating that EDEM1-3 could in fact act as mannosidases and that OS-9 and XTP3B are lectins recognizing Man5-6 oligomannosidic N-glycans, this dogma had to change. Thus, it was proposed that EDEM2 actually trims M9 to M8 followed by the other EDEMs and Man1B1 [14]. Therefore, we expected to obtain a cumulative mannose trimming and accelerated degradation by expressing two mannosidases. However, in this case, the enhanced trimming has not initiated the degradation of neither misfolded tyrosinase, nor NHK. Further investigations are required to explore the role of Man1B1 in regulating the ERAD substrates traffic. However, these data support the notion that the upregulation of EDEM3 and other mannosidases such as Man1B1 is tightly regulated within the ER to support the misfolded protein degradation and protein homeostasis. 

In conclusion, dissecting the molecular determinants of EDEM3, we demonstrate that the GH47 domain provides substrate binding even in the absence of mannose trimming, but requires the intermediate domain for folding. The GH47 domain generates the mannose trimmed substrates, whilst the PA and IDD domains modulate the specific timing of their ERAD pathway within the ER. 

## 4. Materials and Methods 

### 4.1. Sequence Analysis and Modeling of Murine EDEM3

Modeling and sequence analysis involving amino acid distribution, accessibility, secondary structure and intrinsic disorder propensities were assessed as previously described [17,40,41]. Briefly fold recognition was performed with Phyre2 [42] while secondary structure, accessibility, and intrinsic disorder profiles were raised as consensuses based on state of the art predictors as follows: Spot-1D and Spot-Disorder2 [43,44], RaptorX-Property [45], PsiPred and DisoPred [46,47], Jpred, I-Tasser [48], Scratch SSpro [49], Yaspin [50], IUpred [51], and Depicter [52]. Secondary structure prediction consensus was generated via a majority rule voting approach, while intrinsic disorder profiles consensus was built upon averaging the predicted probabilities. Models were built with Discovery Studio suite 3.0 from Accelrys-Dassault Systemes and Modeller v9.17 [53] using the X-ray structure of mouse alpha 1,2-mannosidase (PDB: 1NXC) [54] for the GH47 domain (~30% identity and ~50% similarity) and the structure of an aneurinibacillus aminopeptidase (PDB: 2EK9) for modeling the PA domain (~18% identity and ~33% similarity) (“RCSB PDB - 2EK9: Aminopeptidase from Aneurinibacillus sp. strain AM-1 with Bestatin,” n.d.) Coordinate transfer followed by side-chain optimization were used to build the target sequence regions aligned to the template while insertion loops were generated by sterically constrained Gibbs sampling and optimization using Robosample [55]. Steric conflicts were further relieved by simulated annealing with harmonic restraints on backbone atoms found in helical and beta-sheet structures, followed by energy minimization. The overall models were next subjected to repeated rounds of local and global optimization in NAMD [56] using CHARMM36 force field, followed in an iterative manner till convergence by model quality checks using MolProbity [57]. Predictions of EDEM3 posttranslational modification (PTM) were performed with MusiteDeep [58] and the N-glycans experimentally reported to be attached to the protein core were modelled as described in [59] using structural data from the SAGS database [60,61,62].

### 4.2. Cell Lines, Antibodies, and Reagents

HEK293T and HeLa cells were from the European Collection of Animal Cell Cultures. Rabbit anti-EDEM3 (E8906) and rat anti-HA, clone 3F10 (cat: 12158167001) were from Sigma-Aldrich (St. Louis, MO, USA). Mouse anti-HA.11, clone 16B12 (cat: 901503) was from BioLegend (San Diego, CA, USA). Rabbit anti-α1-anti-trypsin (A0012) was from Dako (Jena, Germany). Mouse anti-tyrosinase (T311) antibody (sc-20035) and mouse anti-MAN1B1 antibody (sc-393145) was from Santa Cruz Biotechnology (Dallas, TX, USA). Rabbit polyclonal antibodies were generated in house for tyrosinase [63] and EDEM3 [64]. Rabbit anti-calnexin (ab22595), rat anti-tubulin (ab6161) and mouse anti-actin (ab8224) antibodies were from Abcam (Cambridge, UK). Endoglycosidase H (EndoH, P0702S) and Peptide-*N*-Glycosidase F (PNG-ase F, P0704S) were from New England Bioland (Ipswich, MA, USA). Kifunensine (sc-201364), MG132 (sc-201260) and all other chemicals were from Santa Cruz Biotechnology unless specified.

### 4.3. Plasmids

pcDNA3.1- EDEM3-HA was a kind gift from N. Hosokawa (IFLMS, Kyoto, Japan). pcDNA3.1-NHK-HA was a generous gift from M. Molinari (IRB, Bellinzona, Switzerland). Soluble tyrosinase (ST) was generated as described previously [31]. Man1B1 sequence was amplified with oIB-315 and oIB-316 from HsCD00040812 plasmid (DNASU, Tempe, AZ, USA) and subcloned into *Xho*I/*Not*I site of pLPCX. 

The full length EDEM3 DNA with HA tag sequence inserted upstream of KDEL sequence was amplified with oIB-288 and oIB-290 and cloned into *Bgl*II/*Not*I site of pLPCX vector. This plasmid was further used as a template for the following plasmids. The DNA sequence of MAN, SP::MAN::HA tag::KDEL (only the nucleotide 1-1515) was amplified by overlapping PCR using primers oIB-288F, -306R, -307F, and -308R. The ∆IDD (missing nucleotides 2374-2709) was obtained by overlapping PCR using the primers oIB-288F, -311R, -312F, -308R. The ∆MAN, without mannosidase domain (missing nucleotides 142-1515) was obtained by overlapping PCR using the primers oIB-319F, -361R, -362F, -290R, and pIB-194. The ∆PA (missing nucleotides 1945-2409) was obtained by overlapping PCR using the primers oIB-319F, -363R, -364F, -290R. All EDEM3 mutation variants were cloned into *Bgl*II/*Not*I site of pLPCX. 

The sequence of the primers used for cloning are described in Table A2, while missing amino acids of EDEM3 are presented in Table A3.

### 4.4. Generation of EDEM3-Knocked out (EDEM3-KO) Cell Line

HEK293T cells were co-transfected with CRISPR/Cas9 (sc-408476) and HDR (sc-408476-HDR) plasmid system from Santa Cruz Biotechnology (Dallas, TX, USA), following provider’s guidelines. At 48 h post transfection, 4 µg/mL puromycin was added to the cell media for clone selection. The cells were maintained in the supplemented media for three passages and further, the puromycin concentration was dropped to 2 µg/mL. Selection efficiency was verified by expression level of EDEM3 by Western blotting. The cells were sorted using a FACS Aria III system (BD Biosciences, San Jose, CA, USA). EDEM3 expression level was tested again by Western blotting and the clone presenting no expression of EDEM3 was further used. 

### 4.5. Generation of HEK293T-MAN1B1 Overexpressing (MAN1B1-OE) Cell Line

The MAN1B1-OE cell line was obtained using a retroviral system. Shortly, cells were incubated for 24 h, with retroviral particles produced in HEK293T after co-transfection of packaging vector and donor plasmid, MAN1B1-pLPCX. After 24h the media was changed and at 48 h post infection the cells were selected with DMEM supplemented with 4 ug/mL puromycin, for four passages. Afterwards the cells were routinely maintained in DMEM supplemented with 2 µg/mL puromycin. 

### 4.6. Cell Culture and Transfection

HEK293T, EDEM3-KO, MAN1B1-OE, and HeLa cells were cultured in DMEM (cat: 31966-021) supplemented with 10% fetal bovine serum (cat: 10270-098) from Gibco (Life Technologies, Paisley, UK) and maintained at 37 °C with 5% CO2. Twenty-four hours post-seeding cells were transfected using 1 mg/mL polyethylenimine (transfection reagent: DNA 2:1 *v*/*w*) or Lipofectamine2000 (Invitrogen-Life Technologies, Paisley, UK). In all experiments the ratio of EDEM3: ERAD substrate was 1:1, unless stated otherwise. Cells were harvested 48 h post-transfection and processed immediately or stored at −20 °C until further processing.

### 4.7. Sucrose Gradient Fractionation

HEK293T cells were processed as described previously [19]. Briefly, they were lysed in a Digitonin-containing buffer (1% Digitonin (*w*/*v*), 50 mM Tris-HCl pH 7.3, 5 mM EDTA and 150 mM NaCl). The lysate was precleared at 14,000× *g* for 30 min and loaded on a continuous 10–40% sucrose gradient. Further, it was centrifugated at 39,000 rpm in anSW41 Ti rotor (Beckman, Brea, CA, USA) for 16 h, at 4 °C. The collected fractions were precipitated with 100% TCA (Sigma-Aldrich, St Louis, MO, USA) and the resulting dried pellets were resuspended in a 4% SDS-containing buffer (100 mM Tris-HCl, pH 7.6). After sonication, equal volumes were separated by SDS-PAGE and the proteins were transferred onto nitrocellulose membrane and probed with anti-EDEM3 antibodies.

### 4.8. Western Blotting

Cells were plated, transfected and lysed in 1% Triton X-100 HEPES buffer (50 mM HEPES pH 7.4, 1.5 mM MgCl_2_, 15 mM NaCl, 1 mM EDTA), containing a mixture of protease inhibitors (Roche, Basel, Switzerland), for 30 min on ice. Equal amounts of post-nuclear supernatants were separated by SDS-PAGE and transferred on a nitrocellulose membrane. The membrane was probed with the appropriate primary antibodies either for 1h at room temperature (RT) or over-night (ON) at 4 °C. After 15 min of washing with phosphate buffered saline (PBS)-0.1% Tween at RT, the membrane was incubated with secondary antibodies coupled with HRP for 1 h at RT. Signal detection was performed by chemiluminescent reaction.

### 4.9. Immunoprecipitation and Western Blotting

Cells were plated, transfected and lysed in 2% CHAPS buffer (50 mM HEPES pH 7.4, 200 mM NaCl) for 30 min, on ice. Post-centrifugation, lysates were incubated with the specified antibodies ON at 4 °C. The immunocomplexes were captured on protein A/G Sepharose (cat. 101041, Invitrogen-Life Technologies, Paisley, UK) for 2 h at 4 °C. The beads were washed two times for 15 min in 0.2% CHAPS and the complexes were eluted with Laemmli buffer 1X in TE buffer (10 mM Tris-HCl, 1mM EDTA) by boiling at 95 °C for 5 min. Samples were further processed as for Western blotting.

### 4.10. Pulse-Chase and Immunoprecipitation

Forty-eight hours post-transfection, cells were starved for 20 min in methionine/cysteine free media (Sigma-Aldrich, St. Louis, MO, USA), pulse labeled for 15 min with 50 mCi of [^35^S]-methionine/cysteine (EasyTag™ EXPRESS^35^S Protein Labeling Mix, PerkinElmer, Waltham, MA, USA) and chased for the indicated time points. Cell lysates (50 mM Tris-HCl pH 8.0, 150 mM NaCl, 1% NP-40) and cell media were incubated with the appropriate antibodies ON at 4 °C for immunoprecipitation of the protein of interest. Immunoprecipitation proceeded as described above. Eluates were separated in reducing or non-reducing conditions and proteins were visualized by autoradiography. For experiments using 10 µM MG132 the inhibitor was added 1 h prior to starvation and maintained during starvation, pulse and chase time points. Band densitometry analysis was performed using ImageJ software (NIH, Bethesda, MD, USA).

### 4.11. Immunofluorescence

HeLa cells were cultivated in DMEM media and transfected with EDEM3 constructs for 48h using Lipofectamine2000. The cells were fixed for 20 min in 1% paraformaldehyde, permeabilized with 0.1% Triton X-100 for 3 min and blocked in 1% BSA for 3 h. Staining with primary antibodies was performed ON, at 4 °C in a wet chamber. The next day, the corresponding Alexa-488-conjugated and Alexa-594-conjugated secondary antibodies were added for 30 min at RT and cells were mounted on coverslips. Images were acquired in a multitrack mode using a Zeiss-LSM 700 (63X, 1.4 NA, oil) confocal microscope with a pinhole of 1.0 Airy units and MBS 488/561 band-pass filters; Manders overlap coefficients were determined for 40 individual cells using the colocalization module of Zen 2010 software (Zeiss, Oberkochen, Germany).

### 4.12. Sample Preparation for LC-MS/MS Analysis

Samples were prepared by proteolysis with trypsin using a previously described in gel-digestion protocol [65]. Briefly, each gel lane was cut into 4–5 slices washed with 40 mM ammonium bicarbonate and acetonitrile (ACN) and subjected to DTT reduction and iodoacetamide alkylation. Gel pieces were incubated overnight at 37 °C with trypsin and the resulting peptides were extracted multiple times with 5% formic acid (FA) and ACN. Samples were then concentrated to dryness and kept at −20 °C until further use.

### 4.13. LC-MS/MS Analysis

Before injection each sample was reconstituted in solvent A (0.06% FA and 2% ACN). EDEM1 affinity-enriched samples were analyzed as described previously [19]. For EDEM3 samples, the obtained tryptic peptides were separated on a 15 cm Acclaim PepMap 100 C18 75 m HPLC column using a 90 min 2–30% solvent B (0.06% FA and 80% ACN) gradient. The eluted peptides were analyzed using an LTQ-Orbitrap Velos Pro instrument (Thermo Fisher) using a data-dependent acquisition method in which the top five most abundant ions (+2 charges or higher) from the survey scan performed in the orbital trap were selected for CID fragmentation in the linear ion trap. Dynamic exclusion was enabled with a repeat count of 1 and repeat duration of 30 s for a 500 list size.

### 4.14. Raw Data Analysis

All the raw LC-MS/MS files were analyzed by searching the data against the human version of the UniProtKB database (41,218 sequences as of 14 December 2019) to which the mouse versions of EDEM1 and EDEM3 were appended. The data was searched using the SEQUESTHT algorithm integrated into Proteome Discoverer v1.4 (Thermo Fisher) with the following settings: 10 ppm/0.5 Da mass accuracy for Orbitrap/linear ion trap scans, trypsin with maximum two missed cleavages as the selected protease, Carbamidomethylation (+57.021 Da) on Cys residues as a static modification and Met Oxidation (+15.995 Da) as a dynamic modification. For false discovery rate (FDR) control of peptide spectrum matches (PSM), the Target Decoy PSM Validator module was activated. All the results were filtered at 1% FDR at PSM level, 5 ppm maximum mass accuracy for the precursor ions and 5% FDR at protein level. Only proteins with at least two unique peptides were further kept. Enrichment ratios were calculated by adding one to all spectral count values.

## Abbreviations

ER	Endoplasmic reticulum
ERAD	Endoplasmic reticulum-associated degradation
ERManI/ManIB1	ER alpha 1,2-mannosidase I
EDEM3-KO	EDEM3 knock-out cell line
LC-MS/MS	Liquid chromatography-mass spectrometry/mass spectrometry
ΔPA	EDEM3 lacking the protease-associated domain
ΔIDD	EDEM3 lacking the intrinsically disordered domain
ΔMAN	EDEM3 lacking the mannosidase like domain
MAN	EDEM3 expressing solely the mannosidase -like domain
ST	Soluble tyrosinase
TYR	Tyrosinase
A1-AT	α1-antitrypsin
NHK	Null Hong Kong variant of α1-antitrypsin
CNX	Calnexin
TUB	Tubulin
MAN1B1-OE	Man1B1 overexpressing cell line

## Appendix A

**Table A1:** Log PSMs values of EDEM3 and EDEM1 in LC-MS/MS analysis.

Accession	Gene Names	Sequence Coverage	Unique Peptides	No. of AAs	MW (kDa)	Log PSMs EDEM3	Log PSMs EDEM1
Q2HXL6	EDEM3	33.08%	23	931	104.6	7.80	0.00
Q925U4	EDEM1	26.38%	15	652	73.7	0.00	6.23

**Table A2:** Detailed sequence of used primers.

Name	Sequence	Attached Sequence
oIB-288F	gatagatctACCATGGGCAAAGCCGGCGGCTGCCG	*Bgl*II, Kozak
oIB-290R	gaagcggccgcTCATAGTTCATCCTTAGCATAATCTGGAACATCATATGG	*Not*I, KDEL, HA tag
oIB-306R	GTTTGTGGTGGAAAGCCAAAGCGGTAACAGATG	
oIB-307F	CATCTGTTACCGCTTTGGCTTTCCACCACAAACtatccatatgatgttccagattatg ctaaggatgaactatg	HA tag, KDEL, reverse complement of oIB-306F
oIB-308R (pLPCX primer)	GGTCCTTGGGGCACCCTGGAAACATC	
oIB-311R	AGAAAGGAGCACCTCCACCTGCTTGTG	
oIB-312F	gcaggtggaggtgctcctttctgacACAAAGGCCCAGCCTATAGACTCCATATTAG	Reverse complement of oIB-311R
oIB-319F (pLPCX primer)	GGACTTTCCTACTTGGCAGTACATCTACG	
oIB-361R	CCTACTCATGGGCGCGGCCC	
oIB-362F	gggccgcgcccatgagtaggCGAAGCATCTCAAAGAAGAACACAACCTC	Reverse complement of oIB-361R
oIB-363	TGGAGGCAGCTGCTGCTCCTTC	
oIB-364	gaaggagcagcagctgcctccaGAAGACCAGCCATCTTCTGAAAATGATTCTC	Reverse complement of oIB-363R
oIB-315F	gatctcgagACCATGGCTGCCTGCGAGGGCAG	*Xho*I, Kozak
oIB-316R	gaagcggccgcTTAAGCATAATCTGGAACATCATATGGATAGGCAGGGG TCCAGATAGGCAGAGG	*Not*I, HA tag

**Table A3:** Missing amino acids of EDEM3 constructs.

Plasmid	Missing aa
MAN	506–931
∆IDD	792–903
∆MAN	48–505
∆PA	649–803
